# Maternal high-fat diet impairs glucose metabolism, β-cell function and proliferation in the second generation of offspring rats

**DOI:** 10.1186/s12986-017-0222-2

**Published:** 2017-11-02

**Authors:** Yan-Hong Huang, Ting-Ting Ye, Chong-Xiao Liu, Lei Wang, Yuan-Wen Chen, Yan Dong

**Affiliations:** 1 Department of Endocrinology, Xinhua Hospital, School of Medicine, Shanghai Jiao Tong University, 1665 Kongjiang Road, Shanghai, 200092 China; 2 Shanghai Key Laboratory of Pediatric Gastroenterology and Nutrition, Shanghai Institute for Pediatric Research, Shanghai, China; 3 Department of Obstetrics, Xinhua Hospital, School of Medicine, Shanghai Jiao Tong University, Shanghai, China; 4 Department of Gastroenterology, Xinhua Hospital, School of Medicine, Shanghai Jiao Tong University, Shanghai, China

**Keywords:** Maternal high-fat diet, Glucose tolerance, Islet function, Weight gain, Transgenerational effect

## Abstract

**Background:**

This study aimed to assess the impact of perinatal high-fat (HF) diet in female Sprague-Dawley rats (F0) on glucose metabolism and islet function in their early life of second-generation of offspring (F2).

**Methods:**

F0 rats were fed with a standard chow (SC) or HF diet for 8 weeks before mating, up to termination of lactation for their first-generation of offspring (F1-SC and F1-HF). F1 females were mated with normal males at the age of week 11, and producing F2 offspring (F2-SC, F2-HF). All the offspring were fed SC diet after weaning for 3 weeks. The glucose level and islet function of F2 offspring were assessed at the age of week 3 and 12.

**Results:**

The F2-HF offspring had a high birth weight and maintained a higher body mass at the age of week 3 and 12, along with an impaired glucose tolerance and lower serum insulin levels compared with the F2-SC. β-cell proliferation was also impaired in the islets of F2-HF rats at the age of week 3 and 12. The pancreatic and duodenal homeobox factor-1 (Pdx1) and Neurogenic differentiation 1 (NeuroD1) expressions were decreased in the islet of F2-HF rats at the age of week 12.

**Conclusions:**

Maternal HF diet during pre-gestation, gestation, and lactation in rats could result in the increased body weight and glucose intolerance in their early life of F2 offspring due to impaired β-cell function and proliferation.

## Background

Progressively increasing incidences of obesity and diabetes become a severe public health issue globally. It was estimated about 40% adult women overweight and 15% obese in 2014. Approximately, 42 million children under the age of 5 were also classified as overweight or obese [1]. Decreased physical activity and increased caloric intake are thought to be the key factors in the development of obesity. More recent studies in rats model, however, demonstrated that maternal high-fat diet during gestation and lactation became a significant risk factor for metabolic abnormalities, such as obesity, impaired glucose tolerance, dyslipidemia, and early-onset nonalcoholic fatty liver disease at their early life of F1 offspring, even while offspring were fed with SC after weaning [2–5].

Previous studies from animal models have shown that maternal undernutrition and intrauterine growth restriction induced a glucose tolerance impairment and obesity in both F1 and F2 offspring [6–8], suggesting that the maternal undernutrition might be a genomic-free transmission across generations via a suboptimal uterine environment, which could result in programmed alterations in organ structure and function. An inadequate diet during F0 pregnancy could affect the intranterine development of the F1 offspring, which might in turn impact the F2 offspring [6, 8].

There is, however, little known regarding the impact of perinatal HF diet exposure on the pancreatic islet function of subsequent generations, i.e. their offspring. Therefore, we aimed to assess the impact of a HF diet during pregestation, gestation and lactation on the glucose metabolism and β-cell function and proliferation in their early life of F2 offspring in rats.

## Methods

### Diet on animal model

**Fig. 1:**
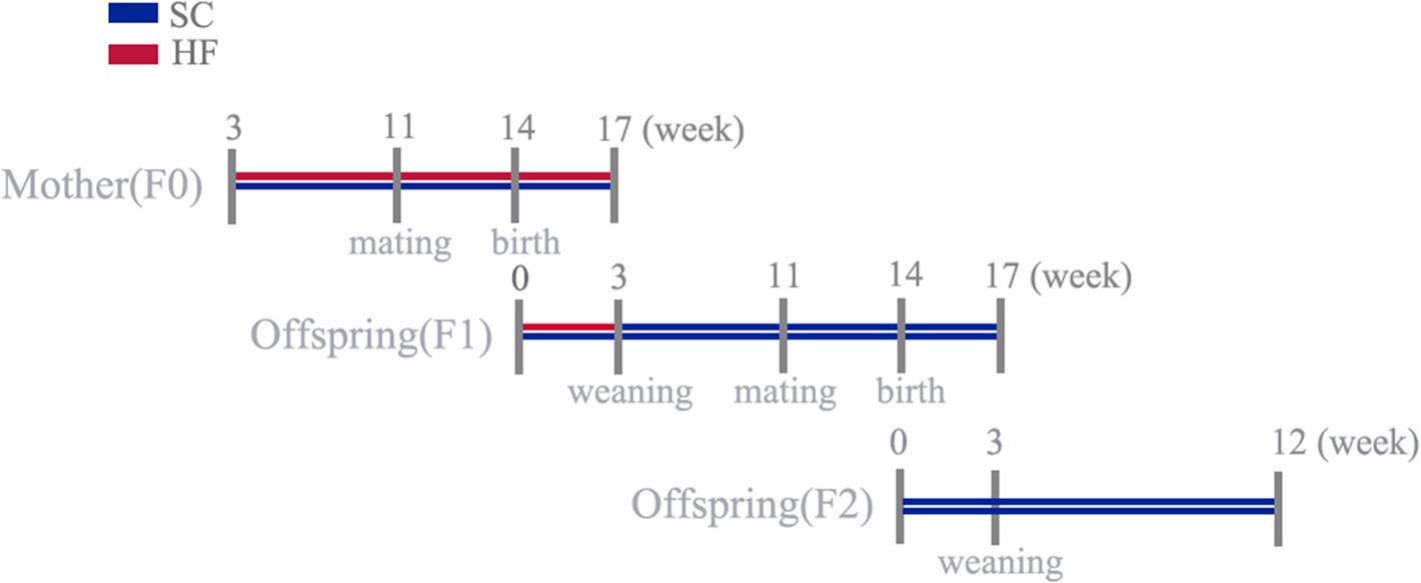
The feeding flow chart for three generations. Figure 1 shows study design. Female Sprague-Dawley rats in SC group consumed a 12.9% fat house chow diet from the age of 3 weeks and throughout the perinatal period, whereas HF group consumed a 45% fat HF diet during the time. Both F1 and F2 offspring were weaned at the age of 3 weeks and fed with SC diet thereafter. The red line indicates the time period in which the dams were fed with the HF diet. The blue line presents the time period in which the SC diet was fed to the control dams, F1-generation and F2-generation offspring. HF, high-fat; SC standard chow

The study protocol was approved by the Animal Use and Care Committee at our institute. Female Sprague-Dawley rats (age of 3 weeks, Shanghai SLRC laboratory animal CO. LTD) were housed in ventilated isolator cages with 12/12 light/dark cycles and a constant temperature of 22 ± 2^o^ C. Animals were given free access to water and food. The dams were fed with SC diet or HF diet for 8 weeks, and were grouped to two, F0-SC, F0-HF, respectively. The HF diet contained (kcal %): fat, 45%; carbohydrate, 29.5%; and protein, 25.5% and the energy density was 4.7 kcal/g, whereas the SC diet was containing (kcal %): fat, 12.9%; carbohydrate, 61.6%; and protein, 25.5%. and the energy density was 3.5 kcal/g, as described previously [9]. Vitamin and mineral contents of both diets were identical (Calcium, 1.24%; Phosphorus, 0.83%; Lysine 1.38%; Methionine and cystine, 0.72%). The females from different litters were mated with males. The dams maintained their respective diets until the end of lactation after pregnancy. Five F1 female offspring in the F1-SC and F1-HF groups were selected and weaned to the SC diet at the postnatal day of 21. Five F1 female offspring from different litters were mated with normal males after 8-weeks to produce F2 offspring. F1 females were fed with the SC diet throughout the pregnancy and the lactation. Six rat pups in F2-SC and F2-HF were selected (1:1 sex ratio), and fed with the SC diet up to 12-weeks after being weaned at 3-weeks. The feeding flow chart was presented in Fig. 1. Note that only F0-HF dams were fed with the HF diet during pregestation, gestation and lactation. The body masses of F0 and F1 dams were measured weekly, and the birth weights of their F2 offspring were documented as well.

### Glucose tolerance tests (GTT)

GTT was performed in F0 and F1 generation before gestation (*n* = 5 /group). It was also determined in two F2 offspring groups at weeks 3 and 12 (*n* = 6 /group). The rats were fasted overnight and injected intraperitoneally with 1.5 g/kg body weight of glucose. Blood was collected from the tail vein at 0, 30, 60, 120 min following the glucose loading. Blood glucose levels were determined by using a handheld glucometer (One Touch Ultra, Johnson &Johnson, New Brunswick, NJ, USA). The glucose tolerence was assessed by calculating the area under the curve (AUC) based on the trapezoid rule (GraphPad Prism version 6.0, La Jolla, CA, USA).

### Blood collection and insulin level

The two groups of F2 offspring were anesthetized with 1% pentobarbital sodium intraperitoneally (40 mg/kg) overnight fasting at weeks 3 and 12. Blood was collected from cardiac puncture and preserved in the tubes, centrifuged at 3000 rpm 4^o^ C for 15 min to isolate the serum. Serum insulin level was determined using enzyme-linked immunosorbent assay (ELISA) (Rat/mouse Insulin ELISA Kit, Alpco, USA). Because there were no international units available for the rats, the amounts of insulin were converted to the units for the human by using the formula:1 mg = 23.1 U. HOMA-β% = 20 × [insulin]/([glucose]-3.5)% and applied to determine β-cell function. [10].

### Immunofluorescence analysis

The pancreas was removed and freeze-clamped immediately, and processed for immunohistostaining and mRNA and protein analyses at the age of week 3 and 12 (*n* = 3 /group). The pancreatic tissues (*n* = 3 /group) were fixed in 10% paraformaldehyde solution and sliced 3 μm in thickness and assessed for immunofluorescence analysis, including immunofluorescence density using insulin (CST, USA) or Ki-67 antibodies (BD, USA) and FITC goat anti-rabbit or Alexa Fluor®594 goat anti-mouse IgG (Jackson, USA). 4,6-diamino-2-phenyl indole (DAPI) (Boster, China) was used to stain the nucleus. Fluorescence was visualized using the microscope (Leica DMI3000B, Germany).

### Pancreatic mRNA genes, Ins1、Ins2、Pdx1、NeuroD1 determination

Total RNA (*n* = 3 /group) was extracted from the pancreas at the age of 12-weeks (n = 3 /group), using Trizol reagent (Takara, Japan) according to the manufacturer’s protocol, and RNA was converted into cDNA by the Power cDNA Synthesis kit (Takara, Japan). Quantitative real-time PCR was performed using ABI7500 system (ABI, Foster City, CA, USA). The mRNA for Insulin 1 (Ins1), Insulin 2 (Ins2), Pdx1, and NeuroD1 in pancreatic tissue were determined. The relative mRNA levels of qRT-PCR products were calculated by normalizing to the level of housekeeping gene 18 s with the 2^-ΔΔCt^ method [11]. Gene-specific primers sequences are listed in Table 1.

**Table 1:** Primer sequences used for mRNA quantification by qRT-PCR

Gene name	Primer sequences (5′ to 3′)	
	Forward	Reverse
Ins1	GTCAAACAGCACCTTTGTGG	CCAGTTGGTAGAGGGAGCAG
Ins2	GTCAAACAGCACCTTTGTGG	TCCAGTTGGTAGAGAGAGCAGA
Pdx1	AAACCGTCGCATGAAGTGGAA	CGAGGTTACGGCACAATCCTG
NeuroD1	GCAAAGGTTTGTCCCAGC	ACGTGGAAGACGTGGGAG
18 s	AAGTTTCAGCACATCCTGCGAGTA	TTGGTGAGGTCAATGTCTGCTTTC

*Ins1* Insulin 1, *Ins2* Insulin 2, *Pdx1* pancreatic and duodenal homeobox factor-1, *NeuroD1* Neurogenic differentiation 1

### Western blot analyses

The pancreatic tissue samples (~30 mg) at the age of 12-week (*n* = 3 /group) were prepared by homogenization in a Radio Immuno Precipitation Assay (RIPA) buffer with protease inhibitors and centrifuged at 12,000 rpm, 4^o^ C for 15 min. The protein concentrations were quantified by modified BCA protein assay (Beyotime, China). 100 μg cell lysates from each sample were separated by 12.5% SDS-PAGE gel and transferred to polyvinylidene fluoride membranes. The membranes were blocked with 5% nonfat dried milk powder in TBST buffer for 2.5 h and were incubated with anti-Pdx1 (Abcam, USA) (1:500), anti-NeuroD1 (CST, Germany) (1:1000) and anti-pNeuroD1 (CST, Germany) (1:1000) overnight at 4^o^ C. Anti-β-actin (Sigma, USA) (1:1000) was used as an internal control. The membranes were developed using ECL reagents (Millipore, Germany) and proteins were finally detected by a chemiluminescence detection system.

### Statistical analyses

Data were expressed as mean ± SE, and statistical analyses were performed using SPSS (version 19.0, IBM, Chicago, IL). Significant differences between SC and HF in each generation were assessed using the independent Student’s *t* test after a Pearson Normality test. *P* value < 0.05 was considered as significant differences between the groups.

## Results

### Body mass and glucose metabolism during pregestation and gestation

F0-HF and F1-HF dams were overweight compared with their corresponding control (F0-SC and F1-SC) before mating (*P* = 0.123, 0.051, respectively). AUC analysis of GTT showed that both the F0-HF and the F1-HF dams had a higher AUC than the F0-SC and the F1-SC (*P* < 0.001, < 0.05, respectively), suggesting that the glucose tolerance was impaired pre-gestation in the F0-HF and the F1-HF dams (Table 2).

**Table 2:** Body mass and glucose metabolism during the pregestation and the gestation in dams

Data	F0-SC	F0-HF	*p*-value	F1-SC	F1-HF	*p*-value
*N*	5	5		5	5	
Pregestational BM (g)	231.7 ± 19.2	248.8 ± 08.7	0.123	219.9 ± 16.1	246.3 ± 20.2	0.051
Pregestational FBG (mmol/L)	4.36 ± 0.49	5.80 ± 0.52^**^	0.002	5.22 ± 0.58	5.08 ± 0.26	0.660
Pregestational GTT-AUC (%)	25.3 ± 2.1	33.3 ± 1.3^**^	<0.001	34.6 ± 2.8	40.8 ± 4.6^*^	0.032
Gestational BM (g)	371.3 ± 23.3	396.5 ± 24.7	0.127	369.6 ± 40.5	399.7 ± 21.6	0.182

*BM* body mass, *FBG* fasting blood glucose, *GTT-AUC* area under the curve of glucose tolerance, *F0-SC* standard-chow fed dams, *F0-HF* high-fat fed dams, *F1-SC* first generation born from standard-chow fed dam, *F1-HF* first generation born from high-fat fed dam

^**^, *P* < 0.01 versus F0-SC

### Body mass in F2 generation

There was no significant difference for the average litter number and the perinatal mortality between the F2-HF and the F2-SC groups. The offspring in the F2-HF had a higher birth mass (*P* < 0.01) and body mass (*P<*0.05), compared with the F2-SC (Table 3).

**Table 3:** Body mass in F2 generation offspring rats (g)

Age	F2-SC	F2-HF	*P* Value
*N*	6	6	
PND 1	5.8 ± 0.2	6.2 ± 0.2^**^	0.008
3-week-old	43.9 ± 5.3	55.7 ± 9.3^*^	0.023
12-week-old	328.8 ± 47.9	391.6 ± 34.4^*^	0.026

*PND* postnatal day, *F2-SC* second generation of grandmother fed with standard-chow diet, *F2-HF* second generation of grandmother fed with high-fat diet

*, *P* < 0.05 versus F2-SC, **,*P* < 0.01 versus F2-SC

### Glucose metabolism in F2 generation

Fasting blood glucose level in the F2-HF was higher than that in the F2-SC at the age of 3 weeks (*P* <0.001). No significant difference between the F2-HF and the F2-SC was observed in fasting blood glucose levels at the age of 12 weeks. AUC of GTT, however, was greater in the F2-HF than in the F2-SC at the age of 3, 12 weeks (*P* < 0.001, 0.05), indicating that the offspring in the F2-HF has a glucose intolerance in their early life. Moreover, the blood insulin level was lower in the F2-HF than that in the F2-SC at the age of 3, 12 weeks (*P* = 0.031, 0.013). The HOMA-β index was also lower in the F2-HF than that in the F2-SC (*P* = 0.013, 0.035; Table 4).

**Table 4:** Serum glucose characteristics in F2 generation

Time	Glucose characteristics	F2-SC	F2-HF	*P* Value
	*N*	6	6	
3-week-old	FBG (mmol/L)	4.6 ± 0.4	6.2 ± 0.5^**^	< 0.001
	GTT-AUC	32.4 ± 1.7	41.5 ± 3.5^**^	< 0.001
	Insulin(μU/ml)	5.3 ± 0.8	4.5 ± 0.1^*^	0.031
	HOMA-β%	108.0 ± 47.8	34.7 ± 7.4^*^	0.013
12-week-old	FBG (mmol/L)	5.3 ± 0.6	5.8 ± 0.3	0.110
	GTT-AUC	37.0 ± 1.4	40.6 ± 3.2^*^	0.039
	Insulin(μU/ml)	12.1 ± 4.1	5.8 ± 0.5^*^	0.013
	HOMA-β%	146.2 ± 81.9	50.8 ± 8.9^*^	0.035

*G* fasting blood glucose, *GTT-AUC* area under the curve of glucose tolerance, *HOMA-β* homeostasis model assessment of insulin secretion index, *F2-SC* second generation of grandmother fed with standard-chow diet, *F2-HF* second generation of grandmother fed with high-fat diet

*,*P* < 0.05 versus F2-SC, **,*P* < 0.01 versus F2-SC

### β-cell proliferation in F2 generation

**Fig. 2:**
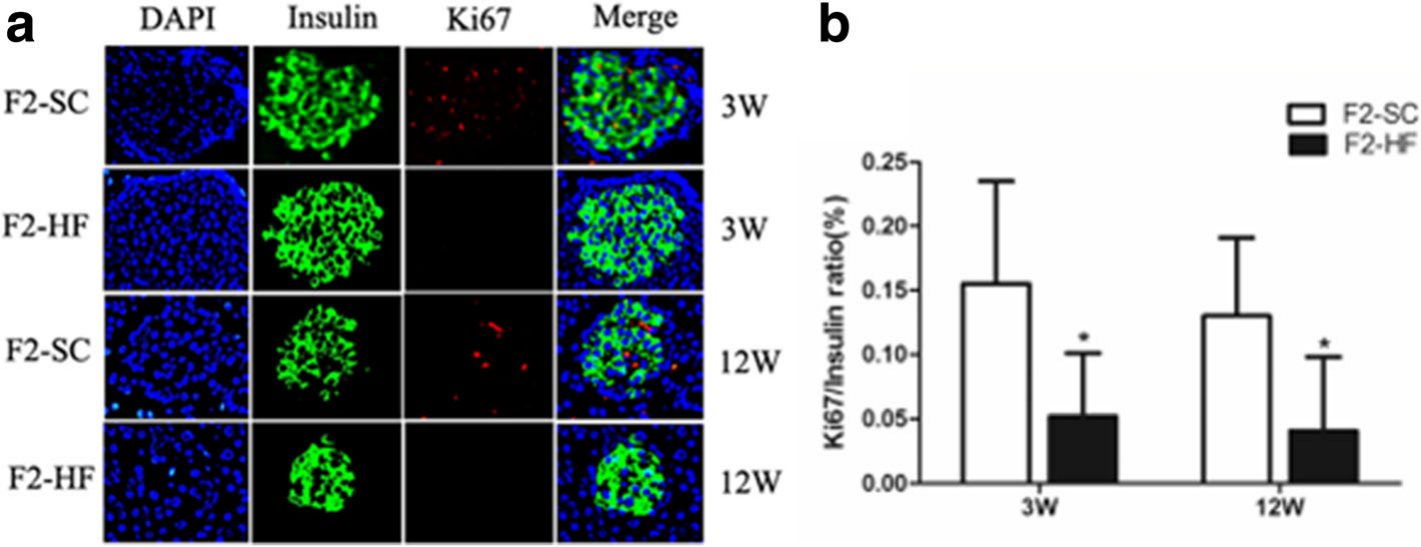
Islets β-cell proliferation in F2 generation at the age of 3 and 12 weeks. **a** Representative immunofluorescence images of insulin and Ki67 expression; (**b**) Quantitative analysis of ki67 positive β-cell /total β-cell (β-cell proliferation frequency). β-cell proliferation frequency in the F2-HF was significantly decreased compared to that in the F2-SC at the age of 3 and 12 weeks. *, *P* < 0.05 versus F2-SC. *N* = 3 per group

An immunofluorescence staining of pancreatic slices was applied to determine β-cell proliferation frequency by marking up the ki67 positive β-cell/total β-cell ratio. The ratio in the F2-HF rats was found to be significantly decreased as compared to the ratio in the control at the age of 3, 12 weeks (*P* = 0.034, 0.036), suggesting that a decreased islet β-cell proliferation in the early life of the F2-HF rats was presented (Fig. 2).

### Gene expression in F2 pancreas

**Fig. 3:**
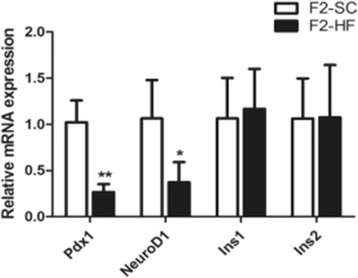
mRNA levels of Pdx1, NeuroD1, Ins1 and Ins2 gene in F2 offspring islets at the age of 12 weeks. The mRNA levels of both Pdx1 and NeuroD1 in pancreatic islets in the F2-HF were significantly lower than those in the F2-SC at the age of 12 weeks. No significant difference was found in the mRNA levels of Ins1 and Ins2 between the F2-HF and the F2-SC. *, *P* < 0.05 versus F2-SC; **, *P* < 0.01 versus F2-SC. *N* = 3 per group

**Fig. 4:**
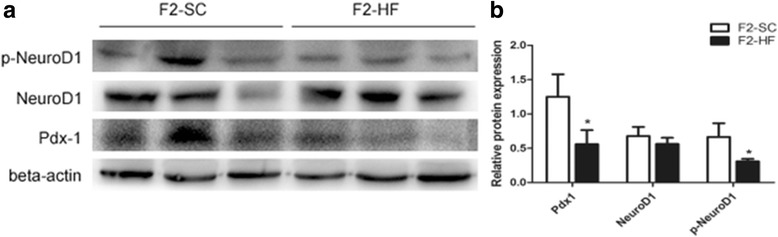
Protein expressions of Pdx1, NeuroD1 and p-NeuroD1 in the F2 offspring islets at the age of 12 weeks. **a** Protein expression levels of Pdx1, NeuroD1 and p-Neuro D1 by western blot analysis; (**b**) the relative protein levels in (**a**). The western blot analyses showed that protein levels of Pdx1 and p-NeuroD1 in pancreatic islets of the F2-HF group were significantly lower than those of the F2-SC group at the age of 12 weeks. No significant difference in protein expression of NeuroD1 was found between these two groups. *, *P* < 0.05 versus F2-SC. *N* = 3 per group

To further understand the mechanisms on a maternal HF diet decreasing islet function in the F2-HF, the insulin mRNA, protein, and other important transcription factors involved in the differentiation and development of β-cells were assessed at the age of 12 weeks. The mRNA levels of the Pdx1 and the NeuroD1 were found to be decreased significantly in the F2-HF with respect to that in the F2-SC (*P* = 0.001, 0.011; Fig. 3). Consequently, Pdx1 protein expression was decreased in the F2-HF as compared with that in the F2-SC (1.25 ± 0.33 versus 0.56 ± 0.21, *P* = 0.036; Fig. 4); NeuroD1 protein expression was slightly reduced in the F2-HF (0.68 ± 0.13 versus 0.56 ± 0.09, *P* = 0.278; Fig. 4). Furthermore, an active form of NeuroD1 in pancreas, phospho-NeuroD1 (p-NeuroD1) protein expression was decreased significantly in the F2-HF as compared with that in the F2-SC (0.66 ± 0.20 versus 0.31 ± 0.04, *P* = 0.040; Fig. 4). There were not significantly differences for the mRNA levels of the Ins1 and the Ins2 gene between the F2-HF and the F2-SC (*P* > 0.05, Fig. 3).

## Discussion

Major findings in this study demonstrated that the trans-generational impact of F0 maternal HF diet on the F2 generation such that the F2 offspring had an increased body weight, glucose intolerance, β-cell function and proliferation impairment, along with a decreased expression of Pdx1 and p-NeuroD1, despite being fed with the SC diet after weaning.

HF diet during the gestation and the lactation can increase body weight and make metabolic abnormalities. A growing number of animal studies recently demonstrated that HF diet during the gestation and the lactation could induce weight gain and metabolic abnormalities, such as pancreatic islet dysfunction in F1 offspring [2–5, 12, 13]. Two studies from Takasaki et al. and Li et al. reported that a continuous HF feeding beyond one generation (derived from both F0 and F1 maternal obesity) promoted body weight increase, hepatic lipid accumulation, and hepatic steatosis in F2 generation offspring [14, 15]. However, both F1 and F2 offspring in these studies were consecutively fed with HF diet, the impacts of F0 maternal HF diet on F2 generation could not be distinguished from the HF diet on F1 and F2 offspring. In this study, only the F0-HF dams were fed with the HF diet, F1 generation and its offspring (F2) were given the SC diet after weaning; this could be expressed as the impacts of F0 maternal HF diet on F2 generation.

The metabolic disorders in the F2-HF offspring could be attributed to the effect of their F0 maternal HF diet. F1-generation from F0 fed with the HF diet in this study showed an increased body mass and an impaired glucose tolerance throughout their early life. Similarly, offspring in F2-HF also presented a higher body mass and impaired glucose tolerance, although F1 dams were fed with the SC diet after weaning and throughout the gestation and the lactation. These results implied that F0 maternal HF diet could induce a trans-generational effect on weight gain and glucose intolerance in F2 generation, similar to the trans-generation in the grandmaternal intrauterine undernutrition [6, 8].

It is worth mentioning that F2-HF offspring had a lower serum insulin level and a lower HOMA-β (%) at the ages of week 3 and week 12 in this study. This was different from the prior studies of maternal HF diet that reported a serum insulin level increase in F2-generation [14, 15]. This difference could be explained by varying diets since they applied HF diet consecutively for three generations while we applied maternal HF diet only in the F0 [14, 15]. HF-feeding could also increase the risk of obesity and obesity-associated disorders, such as hyperglycemia, hyperinsulinemia, insulin resistance, and hepatic steatosis in both animals and humans [2–5, 12].

Understanding the mechanisms on an insulin secretion decrease in F2-HF offspring remains very important in an association of islet β-cell function to the maternal HF diet. We examined the islet β-cell proliferation frequency using the ki67 positive β-cell /total β-cell ratio in the offspring. A significant low ki67/insulin ratios at the age of 3 and 12 weeks was found in the F2-HF offspring, suggesting that F2-HF offspring had a decreased islet β-cell proliferation in their early life. Meanwhile, a corresponding decrease of Pdx1 and p-NeuroD1 expressions was observed in the pancreatic islets, which was reflected by the analytic results from the mRNA and protein expression. More interestingly, Pdx1 and NeuroD1 were pivotal transcriptional factors in the development and differentiation of pancreatic islets. These factors were critical in maintaining the islet β-cell function [16–21]. Pdx1 played a critical role in the survival of the β-cell. The decreased Pdx1 expression in pancreatic islets from type 2 diabetic patients correlated with the decreased insulin expression and decreased glucose-stimulated insulin secretion in human islets [22]. This decreased Pdx1 and p-NeuroD1 expressions in this study was closely associated with reduced β-cell mass in the F2-HF, which led to an impaired glucose tolerance in early life. As the F1-HF dams were fed with the SC diet after weaning, these findings indicated that the F0 perinatal HF diet could transgenerationally impact on the Pdx1 and p-NeuroD1 expression in islet, and also impact on pancreatic islet function in the second generation of offspring.

## Conclusion

In summary, these findings demonstrated that the maternal perinatal HF diet could result in trans-generational abnormalities in persisting for multiple generations. These abnormalities may induce programming retardation in islet β-cell development in the early life of F2 offspring, ultimately leading to an increased body weight with impaired glucose tolerance, a reduced expression of Pdx1 and p-NeuroD1, and a decreased islet β-cell proliferation in the F2 offspring.

## Abbreviations

AUC: Area under the curve
DAPI: 4,6-diamino-2-phenyl indole
ELISA: Enzyme-linked immunosorbent assay
GTT: Glucose tolerance tests
HF: High-fat
HOMA-β: Homeostasis model assessment of insulin secretion index
Ins1: Insulin 1
Ins2: Insulin 2
NeuroD1: Neurogenic differentiation 1
Pdx1: Pancreatic and duodenal homeobox factor-1
SC: Standard chow
